# Leveraging e-Learning technology to enhance pre-service training for healthcare trainees in Ghana: evidence from a pilot project and pointers to policy reforms

**DOI:** 10.1186/s12913-021-07224-3

**Published:** 2021-11-08

**Authors:** Robert Kaba Alhassan, Martin Amogre Ayanore, John-Bosco Diekuu, Emmanuel B. A. Prempeh, Ernestina Safoa Donkor

**Affiliations:** 1 Institute of Health Research, University of Health and Allied Sciences, Ho, Ghana; 2 School of Public Health, University of Health and Allied Sciences, Ho, Ghana; 3 Information Communication Technology (ICT) Directorate, University of Health and Allied Sciences, Ho, Ghana; 4 School of Nursing and Midwifery, University of Health and Allied Sciences, Ho, Ghana

**Keywords:** e-Learning, Pre-service training, Healthcare, Trainees, Pilot study, Ghana, Health policy, Policy reforms

## Abstract

**Background:**

E-Learning solutions are increasingly being advocated to augment existing traditional teaching and learning efforts in health training institutions. Unfortunately, these emerging technologies rarely focus on health sciences education within the context of public universities, hence the need for this study. This project evaluated experiences of healthcare trainees with a pilot e-Learning project (SMART e-Learning project) initiated in one of Ghana’s public universities in 2017. The study used a mixed methods cross-sectional approach among 363 healthcare trainees. Data collection was between 17th October, 2019 to 3rd December, 2019. Data was analysed descriptively and test for variable differences done using Pearson Chi-square and Fisher’s Exact for categorical variables. Wilcoxon Mann-Whitney test was used to test for differences in the Likert scale items. Additionally, rotated varimax factor analysis was performed for the health trainees’ rated satisfaction factors.

**Results:**

Out of 446 respondents who consented to participate in the study, 363 responses were complete and valid, representing a response rate of 81 %. Most frequently used e-learning facilities by healthcare trainees were: writing interim assessments (IAs) (82 %) while the least used function was live chating with faculty (5 %). Challenges associated with the e-learning pilot project were: limited workspace in the pilot computer laboratory (33 %), slow internet/intranet speed (29 %) and limited capacity of teaching and ICT staff to support users (28 %).

**Conclusions:**

There is the need to engage relevant stakeholders at the University, ministries of health and education through policy dialogues to develop statutory e-Learning guidelines for health educational institutions of higher learning to complement existing traditional teaching and learning approaches.

## Background

E-learning approaches are increasingly recognised as potential complementary strategies that could be leveraged to promote efficiency and quality of training for healthcare professionals, particularly in public universities [1–4]. Increasing number of trainees in health training institutions coupled with limited educational infrastructure and other teaching and learning materials [4] make e-Learning solutions compelling complementary options, particularly for resource-constrained countries like Ghana.

Within the Sub-Saharan African region, several e-Learning strategies have been initiated with varying outcomes with respect to efficacy and overall impact on the quality of health sciences education [5–9]. In Ghana, e-Learning innovations have already been piloted in some public universities [4, 10–12], but, there has not been particular focus on health sciences education within the university environment.

Moreover, many previous studies on Ghana [4, 10–12] and elsewhere [13–18] on e-Learning innovations in health education have been limited to practising health professionals already on the field without much attention to pre-service training for health trainees. Albeit the quality of pre-service training offered to health sector workforce has a critical correlation with the quality of health care eventually rendered to clients [1, 11, 12, 19–22], it appears the pre-service component of the entire value chain is relegated to the background notwithstanding its positive impact on health systems in Africa [2, 3]. Likewise, rapid diffusion of mobile telephony has opened up enormous opportunities particularly in the area of health education, making e-Learning solutions potential transformational tools for health sector workforce training and skills development [23–25].

Available reports indicate that access and ownership of mobile phones is on the ascendency in Ghana, like other countries in Africa [23–25]. For instance, in Ghana, total mobile subscription increased by 1.3 % from 36,138,706 in the first quarter of 2016 to 36,613,987 at the end of the second quarter of 2016 [26]. On the other hand, mobile penetration increased marginally from 131.0 % in the first quarter of 2016 to 131.9 % at the end of the second quarter of 2016 [26].

Mobile phone ownership among students in institutions of higher learning is projected to be more in many developing countries like Ghana. In a study by Alhassan [4] it was found that all 233 health trainees interviewed owned a smartphone; however, barely 33 % of them used these smartphones for direct e-Learning purposes while a whopping 88 % of them used the devices for social media communication, particularly WhatsApp.

Important barriers hamper full adoption of these e-Learning innovations in resource limited settings. Key among the challenges is unreliable internet infrastructure which threatens sustainability of these innovations. In addition, in many parts of Africa, internet-based systems suffer from poor connectivity and prohibitive pricing that many public institutions including universities are unable to bear the cost and sustain the financing regimen imposed by telecommunication service providers. In light of these bottlenecks and more, the SMART e-Learning pilot project was initiated to promote quality of pre-service training offered to health trainees in one of Ghana's youngest public universities.

### Overview of the pilot SMART e-Learning project

The SMART e-Learning pilot project entailed provision of learning content (e-books, links to database articles, distribution of course updates); communication from lecturers to students (sharing lecture materials/resources, academic announcements); online/offline assessments (quizzes, assignments, case scenarios, case studies which are all timed & secure, self-test, scoring component), and creation of discussion for a (students can communicate with lecturers & peers, and interact as a class or in groups) for students and teaching faculty. The five (5) implementation phases of the project were: phase 1: baseline feasibility study; phase 2: ICT infrastructure set-up; phase 3: capacity building activities; phase 4: implementation, and phase 5: impact evaluation and scale-up. Details on the SMART e-Learning pilot are published in Alhassan [4].

### Significance and expected outcome

Poor quality of pre-service training for health trainees has been blamed on the increasing intake of students in the health training institutions [4, 19] including universities, coupled with limited number of teaching faculty and educational materials. Smart learning solutions such as e-Learning are therefore promising strategies to augment existing traditional teaching and learning approaches in health training institutions. Additionally, relatively young public universities with limited infrastructure and human resource capacities could leverage e-Learning innovations to promote access to good quality educational materials and professional skills acquisition.

The e-Learning pilot project (called SMART e-Learning Project) therefore aimed at addressing these existing challenges in the context of health sciences education in Ghana. The pilot project incorporates close-range IP Intranet connectivity (WLAN) that is not reliant on internet connectivity for transferring e-Learning materials and services for beneficiary students and staff. These materials and services were made available to end users through their existing mobile devices, free of charge and independent of the quality of internet connectivity on campus. This paper presents outcome of the pilot project among students (also called healthcare trainees) and other beneficiaries in a public university in Ghana solely dedicated to training healthcare professionals.

## Methods

### Study design

This is a cross-sectional descriptive survey aimed to ascertain the personal experiences of beneficiaries of SMART e-Learning pilot project. This design is deemed appropriate because it helps to examine associations between key variables of interest [4].

### Study setting and population

The study was conducted at the University of Health and Allied Sciences (UHAS) in the Volta region of Ghana. At the time of writing this paper, UHAS had six functional schools and two institutes with a population of nearly 5,000 students and about 600 teaching and non-teaching staff. The study sites were two schools at the Ho Campus of the university, namely School of Nursing and Midwifery (SONAM) and School of Allied Health Sciences (SAHS).

SONAM is currently the most populous school constituting over 50 % of the university student population with over 30 teaching and non-teaching staff. SONAM has three departments namely: Nursing, Midwifery and Public Health Nursing. SAHS on other hand has a teaching faculty strength of about 44, and five (5) out of the six (6) departments are currently operational, namely: medical laboratory sciences, physiotherapy and rehabilitation sciences, speech, language and hearing sciences and medical imaging.

The SMART e-Learning pilot project was inaugurated on 24th May, 2018 and followed by series of activities to ensure its successful implementation as described earlier in the background section of this paper. The pilot implementation was done in SONAM and SAHS in the first and second semesters of the 2018/2019 academic year. At the time of the pilot project evaluation, a total of 675 students, faculty and ICT personnel were enrolled on *Moodle* e-Learning platform. In addition, over 15 undergraduate and post graduate courses were mounted on the *Moodle* platform for five departments in SONAM and SAHS. The study participants were mainly in their first, second and third years of their studies at the time of conducting this study.

In the case of SONAM only undergraduate students were enrolled in the pilot e-Learning project while in SAHS the pilot included undergraduate and post-graduate students.

The *Moodle* e-Learning platform was actively used for sharing e-books, videos, and interim assessments (IAs). End of semester examinations were not written via the *Moodle* platform because the university was yet to adopt e-Learning policy for university-wide examinations.

### Inclusion & exclusion criteria

SONAM and SAHS which were beneficiaries of the pilot e-Learning project were included in the evaluation. Conversely, students and staff who did not have an experience with the e-Learning pilot project were excluded.

### Sampling procedure

Sampling strategy was a census approach where all registered students on the pilot project who also met the eligibility criteria were recruited. In the case of SONAM a census of first and second year students from the departments of Nursing, Midwifery and Public Health Nursing who were enrolled in the pilot e-Learning project qualified to participate in the study. Thus, a total sample size of 241 was earmarked for first year  students and 183 earmarked for second year students from SONAM. In the case of SAHS there was a census of 32 students enrolled in the pilot project from the department of medical imaging (Radiology).

### Data collection instrument

The quantitative evaluation tool was in the form of structured questionnaire for students and  was administered electronically using an offline platform (apps.uhas.edu.gh/survey) hosted in the Local Area Network (LAN) of the SMART e-Learning pilot project.

The structured questionnaire administered to students via the electronic platform was made up comprised of nine sections namely Section A (Background information), Section B (Mobile phone ownership and usage), Section C (Experience with the e-Learning pilot project), Section D (Rated satisfaction with e-Learning computer laboratory), Section E (Rated satisfaction with intranet and internet facility), Section F (Rated satisfaction with desk-top computers), Section G (Rated satisfaction with e-Learning content), Section H (Rated satisfaction with faculty), I (Rated satisfaction with ICT staff), and Section J (Suggestions for improvement).

### Validity and reliability

To promote reliability and validity of the data collection instruments, study objectives guided the development of the survey tool. Additionally, there were series of peer reviews and piloting before the tool was implemented. Moreover, scale reliability of the 38 Likert scale items was checked and the average inter-item covariance was 64 while the Cronbach’s alpha scale reliability coefficient was 97%, above the 70% rule of thumb.

### Data collection procedure

Following the pre-test, the quantitative data collection lasted from 17th October, 2019 to 3rd December, 2019. The quantitative data collection tool was administered via an electronic platform where eligible study participants voluntarily consented and took turns to respond to the questions in the e-Learning pilot computer laboratory. The questionnaire took approximately 15 min to administer per respondent.

### Data analysis

Quantitative data was analysed with STATA statistical analysis software (version 12.0). All data sets were coded to anonymize identity of respondents. Descriptive analysis and test for variable differences was done in cross tabulations using Pearson Chi-square and Fisher’s Exact for categorical variables. Wilcoxon Mann-Whitney test was used for the Likert scale items and test for significance set at 95% confidence level. Additionally, rotated varimax factor analysis was performed for the students’ rated satisfaction factors.

### Variables and measures

Main outcome variables of interest for the ordered logistic regression (OLS) were the students’ rated satisfaction with the pilot project based on the factor-analyzed Likert scale items. Bivariate probit regression test was conducted to isolate significant predictors of students’ utilization pattern of the e-Learning programme, while controlling for relevant covariates. The key outcome variables of interest in the regression model were use of e-learning platform for writing interim assessments (IAs); downloading learning materials; uploading class assignments; live chats with faculty; live chats with colleague students, and overall frequency of use of e-Learning resources in a month. The predictive variables after conducting multicollinearity diagnostics were: age of respondent; year of study; gender; school; department, and use of mobile phone for e-Learning purposes (i.e. proxy for information technology (IT) savvy).

Besides gender, other significant predictors of e-Learning utilization patterns were: year of study and use of mobile phone for e-Learning (i.e. proxy for IT savvy). Ordered logistic regression test was further conducted to ascertain the correlates of students’ satisfaction with the e-Learning programme, while controlling for relevant variables of interest. The main outcome variables were five factor-analyzed items on the e-Learning programme (on a five-point Likert scale). The main explanatory variables of interests (in binary outcomes) were: six e-Learning utilization factors by respondents after multicollinearity diagnostics. Co-variates of the model were: age, gender, school, department, year of study, and being IT savvy or not.

### Ethical considerations and institutional approval

 Ethical clearance was given by the Research Ethics Committee (REC) of the University of Health and Allied Sciences on 2nd August, 2019 (clearance number: UHAS-REC A.11[2]18-19). Recruitment of participants in the evaluation was mainly voluntary without coercion after the study protocol was explained to respondents for their individual consent before enrollment into the study. Only participants who provided voluntary informed consent were included in the study.

 Information from respondents was treated confidentially in accordance with the UHAS- REC guidelines for conducting research throughout the various stages of the study. Assurance was given to participants that all information collected from participants will be used mainly for the purposes of this study and not be made available to a third party without their prior consent. All data sets were secured with the project principal investigator.

 Participants were given snack during the data collection to compensate for their time spent in responding to the questions. Even though there were no direct immediate personal benefits to participants, the expected long-term benefit is that findings from this study will help improve on the quality of teaching and learning leveraging opportunities the e-Learning pilot project offers to students and faculty. All study methods were carried out in accordance with relevant guidelines and regulations.

## Results

### Background information

All 446 students who have experienced the pilot e-Learning project were contacted, but 363 of them voluntarily participated representing an overall response rate of 81 %. Majority (91 %) of the respondents were from SONAM and remaining 9 % from SAHS. In terms of the response rate by school and year of study, 100 % of student beneficiaries from SAHS participated, 95 % of second year students from SONAM participated compared to 65 % of participation by first year SONAM students (please see Table 1).

**Table 1:** Return Rate for quantitative data

Category	Target (N)	Sample (n)	Return Rate	Data Collection Period
**Quantitative Data**				17/10/2019–03/12/2019
SONAM (first year)	241	157	65 %	17/10/2019–03/12/2019
SONAM (second year)	172	163	95 %	17/10/2019–03/12/2019
SAHS (all years of study including postgraduates)	32	32	100 %	17/10/2019–03/12/2019

Data source: Field Data by Project Management Team (2020)

The data show that over 90 % of the study participants were students of SONAM while barely 6 % of them were from SAHS. About a third (37 %) of the respondents were nursing students; 31 % were public health nursing students while 25 % of them were midwifery students. Radiology students constituted 6 % of the respondents. In terms of the year of study, 50 % of the respondents were first year students and 48 % were second year students at the time of the conducting the study. In terms gender disaggregation, over a third of the respondents were females and the remaining were males. Out of the 353 valid responses on age, the average age was found to be 21 ± 2 (see Table 2).

**Table 2:** Background information of respondents

Background Characteristics	Frequency (f)	Percentage (%)
**School**		
SONAM	331	91
SAHS	32	9
**Total**	363	100
**Department**		
Nursing	126	35
Public Health Nursing	109	30
Midwifery	91	25
Radiology	32	9
Missing system	5	1
**Total**	**363**	**100**
**Level of study**		
Level 100 (first year)	182	50
Level 200 (second year)	176	48
Missing system	5	1
**Total**	**363**	**100**
**Gender of respondent**		
Female	266	73
Male	97	27
**Total**	**363**	**100**
**Age**	Mean	SD
Obs. = 353	21.2	2.3

**Field Data (2019)**

### Ownership of mobile phones and utilization of e-Learning facilities

Table 3 shows the responses on mobile phone ownership and usage in relation to pilot e-Learning project. It was found that nearly all (96 %) of respondents owned a mobile phone and the type of mobile phone predominantly owned was smartphone; gender and year of study did not significantly show a difference in relation to these parameters. Responses from the study participants show that the most used mobile phone functions were: play/listen to music (35 %), make calls (13 %), intract on social media (7 %) and for academic (11 %) purposes.

**Table 3:** Ownership/usage of mobile phones and e-Learning facility

	Gender			Year of study		
**Response**	**Male**	**Female**	**Total**	**First year**	**Second year**	**Total**
	**f(%)**	**f(%)**	**f(%)**	**f(%)**	**f(%)**	**f(%)**
**Ownership of a phone**						
Yes	93 (26)	262 (72)	**355 (98)**	177 (49)	173 (48)	**350 (96)**
No			**8(2)**			**13(4)**
**Ownership of smartphone**						
Yes	91 (26)	261 (74)	**352 (97)**	174 (48)	173 (48)	**347 (96)**
No			**11(3 %)**			**16(4)**
**Mostly used phone functions**						
Music	28 (8)	100 (28)	**128 (35)**	66 (18)	59 (16)	**125 (34)**
Calls	12 (3)	34 (9)	**46 (13)**	19 (5)	26 (7)	**45 (12)**
Social media	6 (2)	18 (5)	**24 (7)**	11 (3)	13 (4)	**24 (7)**
Academic	12 (3)	28 (8)	**40 (11)**	24 (7)	16 (4)	**40 (11)**
Missing system			**123(34)**			**129(36)**
**Mostly used e-learning functions via mobile phone**						
Write Interim Assessment						
Yes	56 (15)	132 (36)	**188 (52)**	61 (17)	122 (34)	183 (51)*
No	41 (11)	134 (37)	**175(48)**	121 (34)	54 (15)	**175(49)**
Download learning materials						
Yes	41 (11)	96 (26)	**137 (38)**	63 (17)	70 (19)	**133 (37)**
No	56 (15)	179 (49)	**226 (62)**	119 (33)	106 (30)	**225(63)**
Upload class assignment						
Yes	27 (7)	89 (25)	**116 (32)**	35 (10)	79 (22)	114 (32)*
No	70 (19)	177 (49)	**247(68)**	147 (41)	97 (27)	**244(68)**
Live chats with colleagues						
Yes	24 (7)	36 (10)	60 (17)*	31 (9)	29 (8)	**60 (17)**
No	73 (20)	230 (63)	**303 (83)**	151 (42)	147 (41)	**298 (83)**
Live chats with lecturer						
Yes	8 (2)	11 (3)	**19 (5)**	9 (2)	10 (3)	**19 (5)**
No	89 (25)	255 (70)	**344 (95)**	173 (48)	166 (46)	**339 (95)**
**Mostly used e-learning functions via computer lab.**						
Write Interim Assessment						
Yes	86 (24)	212 (58)	298 (82)*	138 (39)	155 (43)	293 (82)*
No	11 (3)	54 (15)	**65 (18)**	44 (12)	21 (6)	**65 (18)**
Download learning materials						
Yes	39 (11)	96 (26)	**135 (37)**	57 (16)	74 (21)	131 (37)*
No	58 (16)	170 (47)	**228 (63)**	125 (35)	102 (29)	**227 (63)**
Upload class assignment						
Yes	30 (8)	91 (25)	**121 (33)**	30 (8)	88 (24)	118 (32)*
No	67 (18)	175 (48)	**242 (67)**	153 (43)	88 (25)	**240 (68)**
Live chats with lecturer						
Yes	9 (2)	9 (2)	18 (5)*	9 (2.5)	9 (2.5)	**18 (5)**
No	88 (24)	257 (71)	**345 (95)**	173 (48)	167 (47)	**340 (95)**
Live chats with colleagues						
Yes	22 (6)	39 (11)	**61 (17)**	30 (8)	31 (9)	**61 (17)**
No	75 (21)	227 (63)	**302 (83)**	152 (42)	145 (41)	**297 (83)**
**Devices used for e-learning**						
Personal laptop	7 (2)	19 (5)	**26 (7)**	16 (4)	9 (2)	25 (7)*
Desk-tops in computer lab	55 (15)	176 (48)	**231 (64)**	128 (35)	101 (28)	229 (63)*
Personal tablet	1 (0.3)	2 (0.5)	**3 (0.8)**	1 (0.3)	2 (0.5)	3 (0.8)*
Personal mobile phone	30 (8)	45 (12)	**75 (21)**	22 (6)	52 (14)	74 (20)*
Missing system			**28 (8)**			**32(9)**
**e-learning utilization**						
5–10 times	14 (4)	20 (6)	**34 (9)**	17 (5)	17 (5)	**34 (9)**
11–20 times	1 (0)	4 (1)	**5 (1)**	4 (1)	0 (0)	**4 (1)**
> 21 times	2 (1)	1 (0)	**3 (0.83)**	2 (0.55)	1 (0.28)	**3 (0.83)**
Missing system			**321 (88)**			**322 (89)**
**e-learning beneficial**						
Yes	66 (18)	179 (49)	**245 (67)**	134 (37)	109 (30)	243 (67)*
No	31 (9)	87 (24)	**118 (33)**	48 (13)	67 (18)	**115 (32)**
Missing system						**5(1)**
**Most beneficial feature**						
Access to learning materials						
Yes	38 (16)	114 (47)	**152 (62)**	67 (28)	83 (34)	150 (62)*
No	28 (11)	65 (27)	**93 (38)**	67 (28)	26 (11)	**93(38)**
Examination score/grade						
Yes	50 (20)	113 (47)	**163 (67)**	95 (39)	67 (28)	**162 (67)**
No	16 (7)	66 (27)	**82 (33)**	39 (16)	42 (17)	**81 (33)**
Live chats with lecturer						
Yes	9 (3.5)	9 (3.5)	18 (7)*	8 (3)	10 (4)	**18 (7)**
No	57 (23)	170 (69)	**227 (93)**	126 (52)	99 (41)	**225 (93)**
Live chats with colleagues						
Yes	20 (8)	18 (7)	38 (16)*	21 (9)	17 (7)	**38 (16)**
No	46 (19)	161 (66)	**207 (84)**	113 (47)	92 (38)	**205 (84)**

Field Data (2019); ***Fisher’s Exact test (p < 0.05)***

The most frequently used e-Learning functions by respondents via their personal mobile phones were writing of interim assessments (IAs) (52 %), download learning materials (38 %), upload class assignments (32 %), live chats with lecturers (5 %) and live chats with colleague students (17 %). It was observed that more females than males used the e-Learning platform for live chats with colleague students (*p* < 0.05). The trend with respect to use of the e-learning platform via the e-Learning computer laboratory were largely similar to usage of personal mobile phones (see Table 3).

Devices used to access the e-Learning facility were desk-tops in the computer lab (63 %), personal mobile phones (20 %), personal laptops (7 %) and personal Tablets. Frequency of utilisation of the e-Learning facility in a month was generally low with 9 % indicating they used the facility 5–10 times and barely 2 % used it 11 times or more in a month. In terms of perceived benefit of the e-Llearning programme, nearly 70 % of them said it was beneficial. Examination score/grade attained during e-Learning assessment was perceived as the most beneficial component of the programme, followed by live chats with colleagues (10 %), access to learning materials (9 %) and live chats with faculty (5 %). More females than males perceived the e-learning programme as beneficial for live chats with colleagues (*p* < 0.05) (see Table 3).

### Personal experiences with the e-Learning pilot project

The experiences of students on the following areas were assessed, namely computer laboratory physical environment and e-Learning inter/intra-net connectivity. In case of the computer laboratory, the highest rated satisfactory condition was the room temperature (mean = 3.6) while the least was physical environment (mean = 3.3) of the computer laboratory.

Assessment score on the e-Learning platform connectivity showed that safety/security (mean = 3.3) was the highest rated factor while the least rated were internet consistency and reliability (mean = 2.7). Ratings of the desk-top computers show that students rated the ease of access to the e-Learning content via the desk-top computers highest (mean = 3.1) while user friendliness was rated the lowest (mean = 2.9). Ratings on the e-Learning content revealed the highest rated components were feedback on written IAs (mean = 3.6) and representativeness of score attained from IAs (mean = 3.6) relative to the least rated component which was ease of access to e-Learning content (mean = 2.9).

Ratings on the role of teaching faculty in the e-Learning programme showed that the highest rated component was supportiveness of staff (mean = 3.2) while the least rated component was video presentations (mean = 2.3). Ratings on the ICT staff revealed similar results where the highest rated components were supportiveness (mean = 3.2), responsiveness to students’ needs (mean = 3.2), friendliness (mean = 3.2) and least rated component was timeliness of response to needs of students (mean = 3.1) (see details in Table 4).

**Table 4:** Experience with e-learning project and its benefits

Response	Statistics		
**Computer lab**	**Obs.**	**Mean**	**Std. Dev.**
Physical environment	350	3.3	1.1
Room temperature	350	3.6	1.2
Furniture/workspace	354	3.4	1.1
Sitting arrangement	351	3.5	1.2
**Connectivity**			
Speed	343	2.9	1.2
Consistency	342	2.7	1.1
Reliability	340	2.7	1.1
Security/safety	338	3.3	1.2
User friendliness	340	3.2	1.2
**Desk-top computers**			
User friendliness	353	2.9	1.2
Ease of access to e-learning content via desk-tops	352	3.1	1.2
**e-Learning Content**			
Relevance	339	3.4	1.1
Timeliness of post	333	3.0	1.1
Ease of access	346	2.9	1.2
Adequacy of learning materials	337	3.0	1.2
Type pf e-learning content	325	3.1	1.1
Ease of submission of assignments	323	3.1	1.3
Feedback on submitted assignment	325	3.0	1.4
Transparency of assignment assessment	319	3.1	1.3
Privacy in assignment assessment	318	3.5	1.3
Representativeness of scores	319	3.4	1.3
Feedback on written IAs	338	3.6	1.4
Transparency in writing IAs	342	3.4	1.3
Privacy in writing IAs	343	3.5	1.4
Representativeness of scores	329	3.6	1.3
**Teaching staff**			
Supportiveness	348	3.2	1.2
Responsiveness to students needs	348	3.0	1.2
Timeliness of response to students needs	342	2.9	1.2
Friendliness	346	3.1	1.2
Live chats	329	2.6	1.3
Video presentations	320	2.3	1.3
**ICT staff**			
Supportiveness	338	3.2	1.2
Responsiveness to students needs	336	3.2	1.3
Friendliness	335	3.2	1.3
Timeliness of response to needs of students	336	3.1	1.2

Field Data (2019); IAs (Interim Assessments); ICT (Information Communication Technology)

### Rated satisfaction with the e-Learning pilot project

Following an un-rotated factor analysis on all the components of the e-Learning programme, six main factors were generated and the summated rated scores compared across gender, school, age and year of study of the respondents. The results show that the component with the most favorable rating was satisfaction with the computer laboratory while the least rated component was satisfaction with teaching faculty. There were statistically significant differences in the ratings in terms of gender, school, age and year of study by the students (see Table 5).

**Table 5:** Rated satisfaction with components of the e-learning pilot project

	Gender		School		Age		Year of study	
**Components**	**Male**	**Female**	**SONAM**	**SAHS**	**≤ 21years**	**≥ 21years**	**First year**	**Second year**
	**Mean (SD)**	**Mean (SD)**	**Mean (SD)**	**Mean (SD)**	**Mean (SD)**	**Mean (SD)**	**Mean (SD)**	**Mean (SD)**
Computer lab (*n* = 344)	3.3(0.9)	3.5(1.0)	3.4(0.9)	3.1(1.0)	3.4(0.9)	3.4(1.0)	3.4(0.9)	3.5(1.0)
Internet connectivity (*n* = 320)	3.1(0.9)	3.0(0.9)	3.1(0.9)	2.8(0.8)	3.0(0.9)	3.1(1.0)	3.1(1.0)	3.1(0.9)
Desk-top computers (*n* = 347)	3.1(1.1)	3.0(1.0)	3.1(1.1)	3.0(1.0)	3.0(1.1)	3.1(1.1)	3.1(1.1)	3.0(1.0)
e-Learning content (*n* = 247)	3.3(0.9)	3.2(1.0)	3.2(1.0)	3.4(1.1)	3.2(1.0)	3.3(1.0)	3.3(1.0)	3.3(1.0)
Teaching staff (*n* = 306)	3.0(1.0)	2.8(1.0)	2.9(1.0)	2.7(0.9)	2.9 (1.0)	2.8(1.0)	2.9(1.0)	2.9(1.0)
ICT staff (*n* = 330)	3.3(1.1)	3.2(1.2)	3.2(1.2)	2.9(1.1)	3.2(1.1)	3.1(1.2)	3.1(1.2)	3.3(1.1)
Overall satisfaction (*n* = 192)	3.3(0.8)	3.1(0.8)	3.2(0.8)	3.2(0.9)	3.2(0.8)	3.2(0.9)	3.2(0.9)	3.2(0.8)

Field Data (2019)

### Perceived challenges with the e-Learning pilot project and suggested areas for improvement

Respondents were also asked to identify experiences with the e-Learning programme in terms of the challenges associated with it. The results show that 33 % of respondents perceived limited workspace at the computer laboratory as a challenge; 29 % perceived speed of internet and intranet (29 %); 25 % each perceived relevance of e-learning content and limited training of faculty (25 %) as challenges. Additionally, 28 % of the respondents perceived limited training of ICT staff as a challenge. In terms of the internet and intranet speed, older respondents (17 %) perceived it as a challenge than the younger category of respondents (12 %) (*p* < 0.05).

**Fig. 1:**
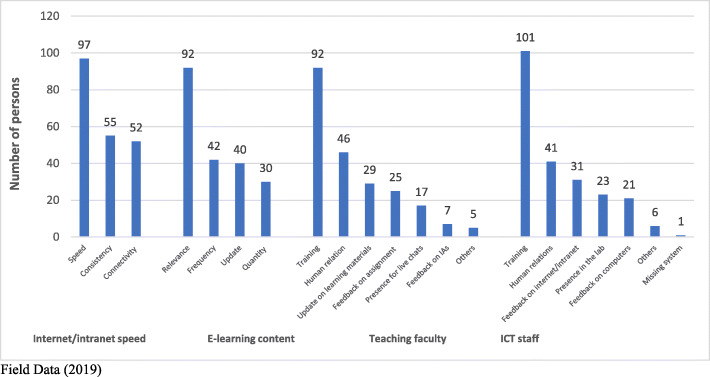
Suggested areas for improvement on e-Learning pilot project

Similarly, more females (20 %) than males (5 %) perceived limited training for teaching staff as a challenge (*p* < 0.05); likewise, more females (23 %) than males (5 %) perceived limited training for ICT staff as a challenge (*p* < 0.05) (see Table 6; Fig. 1). Majority of respondents thus suggested improvements in the areas of internet and intranet speed (*n* = 97), improved relevance in e-learning content (*n* = 92), enhanced training for teaching staff (*n* = 92) and ICT staff (*n* = 101) (see Table 6).

**Table 6:** Challenges with the e-learning project

Response	Gender		School		Age		Year of study		Total
	**Male**	**Female**	**SONAM**	**SAHS**	**≥ 21years**	**≤ 21years**	**First year**	**Second year**	
**Computer laboratory**	**f(%)**	**f(%)**	**f(%)**	**f(%)**	**f(%)**	**f(%)**			**f(%)**
Work space	26 (7)	94 (26)	114 (31)	6 (2)*	46 (13)	74 (20)	60(17)	59 (16)	120 (33)
Lab. room temperature	7 (2)	28 (24)	35 (10)	0 (0)	11 (3)	24 (7)	16 (4)	18 (5)	35 (10)
Furniture	7 (2)	24 (7)	29 (8)	2 1)	12 (3)	19 (5)	14 (4)	17 (5)	31 (9)
User support service	7 (2)	13 (4)	18 (5)	2 (1)	4 (1)	16 (4)	14 (4)	6 (2)	20 (6)
Opening hours	8 (2)	20 (6)	24 (7)	4 (1)	9 (2)	19 (5)	17 (5)	11 (3)	28 (8)
Others	2 (1)	0 (0)	0 (0)	2 (1)	0 (0)	2 (1)	2 (1)	0 (0)	2 (1)
**Internet/intranet**									
Speed	19 (5)	86 (24)	100 (28)	5 (1)	45 (12)	60 (17)*	53(15)	50 (14)	105 (29)
Connectivity	16 (4)	29 (8)	47 (13)	3 (1)	15 (4)	30 (8)	23 (6)	21 (6)	45 (12)
Consistency	11 (3)	39 (11)	40 (11)	5 (1)	12 (3)	38 (10)	26 (7)	24 (7)	50 (14)
Others	13 (4)	20 (6)	29 (8)	4 (1)	7 (2)	26 (7)	20 (6)	13 (4)	33 (9)
**e-Learning content**									
Relevance	18 (5)	74 (20)	87 (24)	5 (1)	36 (10)	56 (15)	47(13)	45 (12)	92 (25)
Update	9 (2)	31 (9)	36 (10)	4 (1)	10 (3)	30 (8)	19 (5)	21 (6)	40 (11)
Frequency	13 (4)	29 (8)	41 (11)	1 (0)	17 (5)	25 (7)	20 (5)	19 (5)	42 (12)
Quantity	5 (1)	25 (7)	29 (8)	1 (0)	10 (3)	20 (6)	13 (4)	17 (5)	30 (8)
Others	9 (2)	12 (3)	16 (6)	4 (1)	4 (1)	17 (5)	14 (4)	7 (2)	21 (6)
**Teaching faculty**									
Training	18 (5)	74 (20)*	88 (24)	4 (1)	31 (9)	61 (17)	46(13)	45 (12)	92 (25)
Human relations	8 (2)	38 (10)	43 (12)	3 (1)	20 (6)	26 (7)	22 (6)	24 (7)	46 (13)
e-Learning materials	7 (2)	22 (6)	28 (8)	1 (0)	9 (2)	20 (6)	14 (4)	14 (4)	29 (8)
Presence for chats	5 (1)	12 (13)	15 (4)	2 (1)	6 (2)	11 (3)	10 (3)	7 (2)	17 (5)
Feedback on assignments	7 (1)	18 (5)	23 (6)	2 (1)	7 (2)	18 (5)	15 (4)	10 (3)	25 (7)
Update on IAs	3 (1)	4 (1)	7 (2)	0 (0)	1 (0)	6 (2)	5 (1)	2 (1)	7 (2)
Others	4 (1)	1 (0)	3 (1)	2 (1)	1 (0)	4 (1)	4 (1)	0 (0)	5 (1)
**ICT staff**									
Training	18 (5)	83 (23)*	98 (27)	3 (1)	41 (11)	60 (17)	30 (8)	25 (7)	101 (28)
Human relations	9 (2)	32 (9)	36 (10)	5 (1)	10 (3)	31 (9)	20 (6)	28 (8)	41 (11)
Present in computer lab	4 (1)	19 (5)	22 (6)	1 (0)	10 (3)	13 (4)	22 (6)	25 (7)	23 (6)
Feedback on computers	5 (1)	16 (4)	20 (6)	1 (0)	6 (2)	15 (4)	16 (4)	15 (4)	21 (6)
Internet and intranet	11 (3)	20 (6)	29 (8)	2 (1)	10 (3)	21 (6)	18 (5)	16 (4)	31 (9)
Others	6 (2)	1 (0)	5 (1)	2 (1)	1 (0)	6 (2)	4 (1)	1 (0)	7 (2)

Field Data (2019)

### Determinants of respondents’ experiences and satisfaction with e-Learning pilot project

Bivariate probit regression test was conducted to isolate significant predictors of students’ utilization pattern of the e-learning programme, while controlling for relevant covariates. The key outcome variables of interest in the regression model were use of e-Learning platform for IAs; downloading learning materials; uploading class assignment; live chats with faculty, live chats with colleague students, and overall frequency of use of e-Learning in a month.

The predictive variables after conducting multicollinearity diagnostics were age of respondent; year of study; gender; school; department, and use of mobile phone for e-learning purposes (proxy for e-learning savvy). E-Learning savvy in this content meant ability of students to independently use smartphone and/or personal computer. The results of the regression analysis showed that being female did not positively associate with students’ likelihood of using the e-Learning platform for writing IAs ([Coef.=-0.49, (95 % CI=-0.82, -1.5), *p* < 0.05]) and uploading class assignments ([Coef.=-0.66 (95 % CI=-1.0, -0.30, *p* < 0.05]) (see Table 7).

**Table 7:** Bivariate probit regression on determinants of e-learning utilization (Boostrap SE)

	Dependent variables (indicators of e-learning utilization)					
**Independent variables**	**Indicator 1**	**Indicator 2**	**Indicator 3**	**Indicator 4**	**Indicator 5**	**Indicator 6**
	**Coef. (95 % CI)**	**Coef. (95 % CI)**	**Coef. (95 % CI)**	**Coef. (95 % CI)**	**Coef. (95 % CI)**	**Coef. (95 % CI)**
**Age**	0.03 (-0.05 0.12)	0.01 (-0.06 0.08)	− 0.002 (-0.08 0.08)	− 0.032 (-0.14 0.073)	− 0.12 (-0.34 0.11)	0.13 (-0.20 0.47)
**Gender**						
Female	− 0.49 (-0.82 − 0.15)*	0.13 (-0.18 0.45)	− 0.66 (-1.0 − 0.30)*	0.18 (-0.29 0.66)	− 0.11 (-1.20. 97)	-7.53 (-71 70)
Male	1.0	1.0	1.0	1.0	1.0	
**School**						
SONAM	− 0.16 (-0.53 0.22)	− 0.22 (-0.54 0.10)	0.06 (-0.35 0.47)	− 0.29 (-0.88 0.302)	0.67 (-0.49 1.83)	0.76 (-0.85 2.37)
SAHS	1.0	1.0	1.0	1.0	1.0	
**Department**						
Nursing	− 0.13 (-0.72 0.47)	− 0.16 (-0.83 0.51)	0.22 (-0.39 0.84)	− 0.17 (-3.85 3.51)	− 0.93 (-2.77 0.90)	-6.42 (-15 15)
Other departments	1.0	1.0	1.0	1.0	1.0	
**Level of study**						
1st year	0.62 (0.27 0.96)*	− 0.31 (-0.63 − 0.001)*	− 0.89 (-1.1 − 0.46)*	0.10 (-0.48 0.67)	0.80 (-0.37 1.97)	-6.81 (-71 70)
2nd year	1.0	1.0	1.0	1.0	1.0	
**Use of phone for e-learning+**						
Yes	1.39 (0.98 1.81)*	0.92 (0.56 1.28)*	0.94 (0.48 1.40)*	0.77 (-1.61 3.15)	0.74 (-0.75 2.23)	-5.62 (-82 82)
No	1.0	1.0	1.0	1.0	1.0	
**Model statistics**	**Bivariate probit Model 1**		**Bivariate probit Model 2**		**Bivariate probit Model 3**	
Log likelihood	-380.50		-238.38		-31.56	
Chi2(1)	5.77		11.01		0.62	
Prob > Chi2	0.0163		0.0009		0.4299	
Wald Chi2(12)	151.18		75.39		7.46	
Replications	50		50		50	
Obs	344		344		39	

Field Data (2019); **Legend**: Indicator 1 (writing IAs); Indicator 2 (downloading learning materials); Indicator 3 (uploading class assignment); Indicator 4 (live chats with faculty); Indicator 5 (live chats with colleague students); indicator 6 (overall frequency of use of e-learning in a month); +Proxy for e-learning savvy

Besides gender, other significant predictors of e-Learning utilisation patterns were year of study and use of mobile phone for e-Learning (proxy of e-learning savvy). As shown in Table 7, it was observed that first year students were more likely to use the e-Learning platform to write IAs relative to second year students ([Coef.=0.62, (95 % CI = 0.27, 0.96), *p* < 0.05]). Conversely, being a first year student negatively correlated with the likelihood of using the e-Learning platform for downloading learning materials [Coef.=-0.31 (95 % CI=-0.63 − 0.001), *p* < 0.05] and uploading class assignments [Coef.=-0.89 (95 % CI=-1.1 − 0.46), *p* < 0.05]. Being e-Learning savvy was also found to have positive association with high likelihood of using the e-Learning platform to write IAs [Coef.=1.39 (95 % CI=(0.98 1.81), *p* < 0.05), download learning materials [Coef.=0.92 (95 % CI=(0.56 1.28), *p* < 0.05) and uploading class assignments ([Coef.=0.94 (95 % CI=(0.48 1.40), *p* < 0.05]) (see Table 7).

As shown in Table 6, ordered logistic regression test was further conducted to ascertain the correlates of students’ satisfaction with the e-Learning programme, while controlling for relevant variables of interest. The main outcome variables were five factor-analyzed factors on components of the e-Learning programme (on a five-point Likert scale). The main explanatory variables of interests (in binary outcomes) were six e-Learning utilisation factors by respondents after multicollinearity diagnostics. Co-variates of the model were age, gender, school, department, year of study, and being e-Learning savvy or not (i.e. ability to independently use smartphone and/or personal computer). The regression model results revealed that satisfaction with internet/intranet reliability [Coef.=4.38 (95 % CI = 1.54, 7.22), *p* < 0.05] and ease of access to the e-learning content [Coef.= 2.15 (95 % CI=-0.01, 4.32)] positively associated with students who used the e-learning platform to write interim assessments (IAs).

Additionally, it was found that student’s satisfaction with privacy of the e-Learning platform for writing IAs is positively associated with students who upload their class assignments via the e-Learning platform ([Coef.=3.63 (95 % CI=-0.06, 7.32), *p* < 0.05]). It was also revealed that students who had live chats with faculty via the e-Learning platform were less likely to be satisfied with the internet/intranet security and safety [Coef.=-2.31 (95 % CI=-4.39, 0.23), *p* < 0.05]; internet/intranet reliability [Coef.=-3.37 (95 % CI=-5.74, -1.00), *p* < 0.05] and ease of access to the e-Learning platform ([Coef.=-3.79 (95 % CI=-6.39, -1.20), *p* < 0.05]). Students who reported they used the e-Learning facility for 10 times or less in a month had a higher propensity of expressing confidence in the privacy of the e-Learning platform for writing IAs ([Coef.=-3.12 (95 % CI=-6.14, − 0.09), *p* < 0.05]).

Covariates which were significantly associated with respondents’ satisfaction level were age, school, department and year of study of the student. Older respondents ([Coef.=-0.47 (95 % CI=-0.79, − 0.15), *p* < 0.05]), being a general nursing student ([Coef.=3.18 (95 % CI = 0.23, 6.14), p < 0.05]) and first year student ([Coef.=-6.08 (95 % CI=-9.93, -2.23), *p* < 0.05]) were significantly associated with the satisfaction levels with privacy of the e-Learning platform for writing IAs. Being a student of SONAM relative to SAHS was also found to be significantly associated with positive experience with reliability of the intranet/internet ([Coef.=2.44 (95 % CI = 0.42, 4.47), *p* < 0.05]) (see Table 8).

**Table 8:** Ordered logistic regression on determinants of students’ experiences with the e-Learning project

	Dependent variables (factor-analyzed satisfaction factors)				
**Independent variables**	**Factor 1**	**Factor 2**	**Factor 3**	**Factor 4**	**Factor 5**
	**Coef. (95 % CI)**	**Coef. (95 % CI)**	**Coef. (95 % CI)**	**Coef. (95 % CI)**	**Coef. (95 % CI)**
**Age**	− 0.21 (-0.49 0.06)	− 0.16 (-0.44 0.12)	0.12 (-0.17 0.40)	− 0.47 (-0.79 − 0.15)*	0.03 (-0.25 0.30)
**Gender**					
Female	0.62 (-1.29 2.54)	1.67 (-0.35 3.70)	0.52 (-1.48 2.51)	0.24 (-1.84 2.32)	1.66 (-0.59 3.91)
Male	1.0	1.0		1.0	1.0
**School**					
SONAM	0.13 (-1.40 1.67)	0.70 (-1.14 2.54)	2.44 (0.42 4.47)*	-1.81 (-3.91 0.28)	1.11 (-0.68 2.89)
SAHS	1.0	1.0		1.0	1.0
**Department**					
Nursing	2.20 (-0.64 5.04)	1.78 (-1.07 4.63)	-1.98 (-5.05 1.08)	3.18 (0.23 6.14)*	0.98 (-1.99 3.94)
Other departments	1.0	1.0		1.0	1.0
**Level of study**					
1st year	-1.47 (-3.67 0.73)	− 0.40 (-2.72 1.92)	0.29 (-2.16 2.75)	-6.08 (-9.93 -2.23)*	0.66 (-1.91 3.22)
2nd year	1.0	1.0		1.0	1.0
**Use of phone for e-learning**					
Yes	− 0.73 (-3.12 1.67)	0.162 (-2.20 2.52)	− 0.02 (-2.32 2.28)	1.13 (-1.17 3.44)	− 0.36 (-2.73 2.02)
No	1.0	1.0		1.0	1.0
Utilization of e-learning	0.68 (-0.94 2.30)	0.07 (-1.64 1.79)	− 0.42 (-2.18 1.34)	1.74 (-0.25 3.72)	− 0.30 (-2.19 1.60)
Write Interim Assessments	1.06 (-0.82 2.93)	0.90 (-1.12 2.92)	4.38 (1.54 7.22)*	1.36 (-0.89 3.61)	2.15 (-0.01 4.32)*
Download learning materials	− 0.99 (-3.30 1.33)	0.25 (-2.19 2.70)	-1.45 (-4.21 1.31)	-2.60 (-5.56 0.36)	1.04 (-1.72 3.79)
Upload class assignments	1.67 (-1.04 4.38)	2.62 (-0.26 5.50)	1.37 (-1.56 4.30)	3.63 (-0.06 7.32)*	1.89 (-0.98 4.76)
Live chats with faculty	− 0.12 (-1.87 1.64)	-2.31 (-4.39 0.23)*	-3.37 (-5.74 -1.00)*	1.26 (-0.82 3.33)	-3.79 (-6.39 -1.20)*
Used facility ≤ 10times in a month	− 0.64 (-2.75 1.47)	− 0.07 (-2.27 2.14)	− 0.58 (-2.87 1.71)	-3.12 (-6.14 − 0.09)*	-1.02 (-3.26 1.23)
**Model statistics**	**Model 1**	**Model 2**	**Model 3**	**Model 4**	**Model 5**
Log likelihood	-49.01	-40.18	-41.06	-42.94	-44.04
LR chi2(12)	9.84	15.15	22.91	22.21	19.99
Prob > chi2	0.6301	0.2333	0.0285	0.0352	0.0672
Pseudo R2	0.0912	0.1586	0.2181	0.2055	0.1850
Number of obs	39	38	38	38	38

Data Source: (Field Data, 2019); Legend: factor 1 (Teaching faculty responsiveness to students’ needs); factor 2 (Internet/intranet security and safety); factor 3 (Internet/intranet_ reliability); factor 4 (Content privacy in writing IAs); factor 5 (Content ease of access)

Field Data (2019)

## Discussion

The project sought to evaluate experiences with a SMART e-Learning pilot project implemented in 2017. A total of 363 (81 %) of the direct beneficiary students of the pilot SMART e-Learning pilot project participated in the evaluation study. Findings of the evaluation revealed that in terms of readiness for an eLearning pilot project deployment, mobile phone ownership was the predominant indicator with 96 % of the study participants indicating their owned a mobile phone that was smart. This revelation reflects the national picture of mobile phone penetration and adoption in Ghana [26] and similar developing countries [4, 15, 20]. Routine usage of the mobile phones by students was however predominantly for entertainment purposes other than academic, similar to studies on the subject matter in other countries [4]. This observation thus, presents an opportunity for institutions of higher learning to leverage the existing potential among students to redirect the usage pattern of smartphones, in particular, for academic related activities to complement existing traditional teaching and learning approaches.

In terms of usage of mobile phone for the implemented e-Learning pilot project, it was found that more than half of the students used their mobile phone devices for writing interim assessments (IAs) on the e-Learning platform. Other e-Learning functions used by students via their mobile phones were downloading learning materials and uploading class assignments. Perhaps, limited capacity of the students, teaching faculty and ICT staff on the *Moodle* at the time of pilot programme might have accounted for the seemingly under-utilisation of the e-Learning resource. Qualitative interviews with some teaching faculty and ICT staff corroborated this conclusion since these stakeholders acknowledged capacity deficits on the *Moodle* platform in the early days of its deployment. Empirical studies in Ghana [4, 19] and other developing countries [6–18, 23–25] on e-Learning innovations for health trainees have also pointed to similar concerns of limited human resource expertise as an existing challenge to e-Learning programme implementation in Africa and other continents with limited resources.

In terms of personal experiences with the e-Learning pilot project, it was found that physical environment of the e-Learning computer laboratory was attributed to reasons users were less satisfied with the e-Learning project. Speed, ease of access and perceived relevance of the e-Learning content were the least rated assessment areas of the *Moodle* platform by students. Individual in-depth interviews conducted among ICT staff, teaching faculty and university managers expressed similar concerns particularly, limited physical infrastructure alluded to by the students. These responses appear to confirm the existing challenge faced by the university in terms of limited ICT infrastructure similar to constraints experienced by many government institutions when it comes of executing the national ICT agenda for Accelerated Development policy launched in 2003 [26].

Studies conducted in other countries in Africa [6–18, 23–25] corroborate these concerns indicated by the beneficiary students of the e-Learning pilot project. In light of these concerns, dedicating ring-fenced budget for ICT infrastructural development by university management would be an important intervention to improve on the existing infrastructural deficits.

Nonetheless, it was observed that beneficiary students had some confidence in the e-Learning pilot project especially in terms of timely feedback on their performance on written examinations via the *Moodle* platform and transparency/representativeness of the grades obtained because of the instantaneous display of examination scores upon completion of an examination. 

Several studies in the past have emphasised the unparallel advantages and benefits of e-Learning teaching and learning environment over the traditional approaches [9–18, 23]. Indeed many studies have advocated for a paradigm shift from mainstream conservative teacher-centred approach to student-centered blended learning due to the afore discussed benefits in pre-service training of healthcare trainees [6, 15, 19, 25].

Findings from this study demonstrates the need for institutions of higher learning, particularly mandated to train healthcare professionals, to take advantage of the exiting opportunities in e-Learning and at the same time addressing constraints and potential threats to sustainability of e-Learning innovations. Among key immediate interventions include working with local telecommunication companies to provide dedicated seamless internet bandwidths to health training institutions of higher learning. This intervention will help address the challenge of slow internet speed for academic activities.

Additionally, there is the need to build the capacity of teaching faculty and ICT staff on e-Learning content development to enhance the quality delivery to students. It was observed that many lecturers simply uploaded teaching slides and pdf documents that were not crafted to meet the needs of students. Many useful features of the e-Learning pilot project were highly underutilized or not used at all. Consistent training in e-Learning content development for all relevant stakeholders should therefore form part of the entire value chain of e-Learning innovations to make them relevant and fit-for-purpose to users.

Limited budget space for additional ICT related expenditure has been a major challenge of many public institutions of higher learning [26]. Consequently, many of these institutions are unable to implement the lofty e-Learning innovations already in existence. National agenda for e-Learning is therefore imperative, especially drawing lessons from the COVID-19 pandemic that virtual ICT innovations are necessary complementary teaching and learning strategies that institutions of higher learning must embrace if they have to remain relevant and competitive in the 21st century health education landscape.

 At the institutional level, it imperative for UHAS to initiate multi-stakeholder dialogues on institutionalising e-Learning as a recognised and lawful teaching and learning method. Developing an e-Learning policy guideline for the university will be an important starting point to consolidate the existing initiatives on e-Learning. Lessons and success stories of other universities within and outside Ghana could guide the process of developing university level e-Learning policy guidelines.

More importantly, scale-up of the current pilot e-Learning project demands that important dynamics of gender, age, students and faculty readiness for e-Learning solutions are considered. These conditions are important because they significantly influence the successful implementation or otherwise of e-Learning innovations as demonstrated in this study. For instance, it was found in this evaluation that more males than females were more likely to utilise the e-Learning resources (*p* < 0.05). Students who often used their mobile phones for e-Learning related activities were also more likely to utilise the e-Learning pilot project (*p* < 0.05). Likewise, elderly students and lecturers were more likely to have challenges adopting to e-Learning innovations relative to their younger colleagues. These observations are similar to studies by Barker et al. [9] and Voutilainen et al. [13] where gender, age and ICT savvy levels associate positively with adoption of e-Learning solutions.

Providing conducive work environment for students, teaching and ICT staff is another strategy to enhance motivation levels for effective e-Learning project implementation as mentioned by Harandi [6]. Even though industrial psychologists generally posit that providing financial incentives do not always guarantee worker motivation, financial incentives such as additional duty and responsibility allowances for frontline staff involved in e-Learning implementation should be incorporated into e-Learning policy documents to promote commitment to assigned duties. Likewise, non-financial incentives such as creating opportunities for capacity building through personal development short courses are proven intrinsic motivational strategies for workers in many industries, including the health sector [27].

Finally, setting up institutional structures for e-Learning management is essential for effective and sustainable implementation of e-Learning project innovations. For instance, clear cut reporting lines and chain of command promotes sanity in the management of e-Learning set-ups and engenders trust and confidence in the system relative to *ad hoc* structures and rules of engagement. Similarly, it emerged from the study that intra-institutional level collaboration and engagements were not optimal during the pilot e-Learning project implementation. This observation appeared to have induced some level of apathy among stakeholders. Dillenbourg [28] argued stakeholder apathy does not promote collaborative learning approaches. Consequently, institutional level work ethics and chain of commands should be clearly stated and unclear pathways addressed ahead of scale-up plans for a university-wide e-Learning strategy.

Even though many health systems have accelerated significantly in terms of e-Learning and mHealth technologies, knowledge levels and adoption of these technologies by students and health professionals in practice remain moderately low. A university-based survey by Peprah et al. [29] among 963 undergraduate students in a public university in Ghana found that prevalence of the use of mHealth stood at 51 % suggesting the positive contribution of these innovative interventions are yet to be fully harnessed. In a similar study by Peprah et al. [30] on leveraging mHealth to lessen barriers to healthcare in rural Ghana, it emerged that even though perceived benefits of technological innovations were high illiteracy, language barrier, trust, quality of care, and poor mobile network connectivity limited their full use. These revelations therefore call for broad stakeholder engagements among telecommunication service providers, health system players and institutions of higher learning to fully maximise the potential of e-Learning innovations for pre-service training of healthcare professionals.

### Recommendations for future research

Based on the current findings and research limitations, it is recommended future research endeavour explores perspectives of practising healthcare professionals on adaptation to e-Learning and mHealth innovations beyond the heath trainees.

Also, future researchers are encouraged to compare experiences and exposure of health trainees in the universities with nursing, midwifery and allied health training colleges to ascertain the potential differences.

Finally, future researchers interested in the topic area could extend the study sites to other universities across the coastal, middle and northern zones to fully appreciate the dynamics in terms of the geopolitical differences in other regions since this study was mainly conducted in one out of the sixteen (16) administrative regions of Ghana.

## Conclusions

It was found from the study that most users of the e-Learning pilot project are satisfied with the programme but pointed out challenges of space, content and capacity of teaching and ICT faculty to effectively execute the project. Also, male, older and nursing students expressed better satisfactions with the e-Learning pilot project relative to other categories of users. Additionally, predominantly used functions of the e-Learning pilot project were writing of IAs, downloading of lecture materials and upload of class assignments. Live chats with colleagues and lecturers were the least utilized functions.

The evaluation findings provide empirical evidence on upscaling prospects and sustainability threats of the pilot e-Learning project in UHAS. Initial evidence demands policy dialogue at the University senior management level and other relevant stakeholders on the possibility of developing statutory guidelines and strategies on e-Learning for the University to complement existing teaching and learning approaches. This intervention will improve on the standard of teaching and learning in the university and ultimately help guarantee quality of trained health professionals for the health sector in Ghana and beyond. In conclusion, e-Learning pilot project innovation is a promising complementary approach for teaching and learning and demands policy consideration by university management for scale-up and sustainability.

## Limitations

The study is not without limitations. First, the pilot project was executed in only two out of seven schools in the University. The feedback might not necessarily reflect the general University picture. Nonetheless, since the student and faculty population of these two schools constitute over 50 % of the University population, at the time of this evaluation, the views expressed potentially represent the general university dynamics.

Also, responses from the stakeholders were largely subjective based on their personal experiences which could not be independently verified to substantiative the veracity or otherwise of the claims. However, piloting the data collection tools and establishing a Cronbach’s Alpha of over 80 % demonstrates a high internal validity of the quantitative tool used for the student survey.

Finally, implementation of the pilot eLearning project was less than one academic year which perhaps did not allow the beneficiaries of the project to sufficiently experience the benefits and challenges associated with it. Perhaps a longer pilot project implementation could have yielded different feedback from the current findings. The findings are nonetheless compelling enough to inform policy decisions on a possible e-Learning policy strategy for the University.

### Policy recommendations

In light of the findings of the pilot e-Learning project evaluation, the following policy recommendations are suggested:

1. University management should commit resources for e-Learning by building the requisite physical and human resource capacity for information communication technology (ICT) in the University.
2. As a matter of urgency, University management in consultation with relevant stakeholders should develop University-wide policy document on e-Learning.
3. Schools, Institutes and Directorates of the University should leverage COVID-19 situation to continually orient teaching and non-teaching staff and students on e-Learning as part of annual students and staff orientation routine.
4. University should make e-Learning mandatory in the teaching and learning value chain through established standard operating procedures (SOPs).
5. As part of the scale-up plan for the pilot project, which has so far proven beneficial, the University should consider rolling out pilot distance modular programme for Sandwich students based on lessons learnt from this pilot project. This move will help reduce congestions on campus by leveraging virtual teaching and learning resources of the e-Learning platform. More revenue is likely to accrue to the University since more students can be taken in virtual classrooms at a relatively lower operational cost without compromising quality of teaching and learning.

## Data Availability

All data generated or analysed during this study are included in this published article.
